# Phase II monitoring of process variability in multichannel profiles

**DOI:** 10.1371/journal.pone.0337707

**Published:** 2025-12-12

**Authors:** Zahra Jalilibal, Amirhossein Amiri, Orod Ahmadi

**Affiliations:** 1 Department of Industrial Engineering, Faculty of Engineering, Shahed University, Tehran, Iran; 2 Department of Industrial Engineering, Faculty of Engineering, Kharazmi University, Tehran, Iran; University of Hamburg: Universitat Hamburg, GERMANY

## Abstract

Monitoring process variability utilizing profile data remains a significant challenge in statistical process monitoring (SPM), especially in the context of multichannel profiles. Detecting shifts in the covariance matrix of a multivariate normal process is crucial for this purpose. The complexity increases notably in high-dimensional processes because of the large number of variables and limited sample sizes. Typically, monitoring changes in the covariance matrix assumes that only a few elements deviate simultaneously from their in-control values. This study introduces a new approach for monitoring the covariance matrix in Phase II for multichannel data. The suggested approach incorporates exponentially weighted moving average (EWMA) control chart with multichannel functional principal components analysis (MFPCA) to derive proposed statistics. Simulation results represent the effectiveness and performance of the suggested approach, highlighting its superior performance in average run length under various shift patterns.

## 1. Introduction

In the current competitive marketplace, companies are increasingly prioritizing numerous process quality characteristics to sustain their market share, improve customer satisfaction, and outperform competitors. The recent advancements in data acquisition technologies have enabled the gathering of extensive information on these quality characteristics. However, analyzing data from processes with a large number of quality characteristics demands significant time and cost. Collecting large samples in this context presents two primary challenges: (1) it imposes substantial costs on manufacturers, and (2) in manufacturing systems with low production speeds, it is impractical to wait for an adequate sample size. As a result, quality practitioners often face situations where the number of process quality characteristics exceeds the sample size, a condition known as “high-dimensionality” [1,2].

Today, manufacturing entities extensively make use of cutting-edge technologies to automatically collect and inspect data; such an approach yields considerable quantities of information as high-dimensional data streams are obtained from several sensors by which the process is illustrated. Accordingly, the chance to monitor online processes and detect anomalies is offered by a sensor [3]. Nevertheless, the development of the online system for monitoring as described above requires dealing with multiple challenges. Significant extents of real-time, sophisticated data have become accessible as a result of innovative sensing technologies present within multifaceted systems of manufacturing. Even though such data offer extraordinary possibilities for monitoring and enhancing the quality of products or the efficiency of processes, they may lead to considerable difficulties regarding the traditional SPM methods as well. This specific form of quality is defined as a response variable’s functional connection to one or more explanatory variables, which varies from univariate or multivariate quality characteristic usually, referred to in the related literature across a large number of SPM applications. Notably, such functional relation is frequently termed as a profile while the profiles’ SPM problem is referred to as profile monitoring [4].

Multi-channel profile monitoring has a wide range of real-world applications across several industries, where data is collected from multiple sensors or channels to monitor and control complex processes. Multi-channel profiles refer to datasets where multiple sensors or data streams capture various aspects of a process or system simultaneously. Analyzing these profiles allows for a comprehensive understanding of complex systems, leading to improved process monitoring and fault detection. Several studies have explored the applications of multi-channel profile analysis across different domains: In advanced manufacturing, multiple sensors are deployed to collect real-time data on various process variables. The integration of these multi-channel profiles facilitates effective process monitoring and fault detection. For instance, in forging processes, sensors measure forces exerted on dies to detect missing parts or anomalies that could lead to defective products or equipment damage [5]. In ultrasonic metal welding, multiple sensors monitor parameters such as vibration and temperature to ensure weld quality. Analyzing multi-channel profiles from these sensors enables the detection of subtle changes in the welding process, facilitating early identification of potential defects [6]. Multi-channel profiles are also utilized in image processing, particularly in hyperspectral imaging, where each pixel contains information across multiple spectral bands. Morphological operations extended to multi-channel data allow for effective feature extraction and classification in remote sensing applications [7]. In the paper by [8], multi-channel profiles are applied to the statistical monitoring of image data using multi-channel functional principal component analysis (MFPCA). This approach enables the simultaneous analysis of multiple image channels, such as color, texture, or spatial features, improving anomaly detection and quality control in image-based systems.

Monitoring multivariate process variability is crucial for the effective implementation of statistical quality control (SQC). In real-world applications, abnormal process variabilities are often linked to a decline in the overall quality of manufactured products [9]. With advancements in production technologies, which are frequently characterized by numerous quality attributes, there has been an increased focus on multivariate statistical process monitoring. Over past years, various multivariate techniques have been extended to monitor changes in process variability. A usual characteristic of these techniques is their reliance solely on information derived from process data [6,10].

Within the broader context of SPM, the concept of a change point plays a critical role in identifying process shifts. A change point refers to a specific time at which the statistical properties of a monitored process—such as its mean, variance, or correlation structure—undergo a sudden and sustained change. Detecting this change point enables practitioners to distinguish between “in-control” (stable) and “out-of-control” (shifted) process conditions. Change-point methods extend traditional control charts, such as Shewhart, CUSUM, and EWMA charts, by explicitly estimating both if and when a permanent shift occurs rather than merely signaling an out-of-control condition. For instance, [11] proposed a change-point control chart based on the sum of squared ranks to monitor production lead time, effectively incorporating the change-point mechanism into SPC. Similarly, [12] developed fuzzy shift change-point algorithms to estimate the time of process mean shifts in control-chart applications. Such approaches provide robust frameworks for enhancing sensitivity and diagnostic capability in complex manufacturing systems.

In SPM, monitoring the quality characteristics (process or product) is essential for prosperous quality development and the reduction of variability in process. Frequently, monitoring process variability involves detecting a deterioration in the correlated quality characteristics, that are represented by the covariance matrix of multivariate quality data. To monitor the process covariance matrix, the sample covariance matrix is commonly utilized as an estimate of the covariance matrix.

Profile monitoring has attracted considerable academic attention as a topic of research due to its significance. The related literature in this regard can be categorized as the following: (i) Profiles that make use of parametric models (linear and nonlinear); subsequently, the monitoring of profile parameters is carried out based on estimations obtained from the profile data [1,13–16]. (ii) The second-- category involves the application of nonparametric models such as nonparametric smoothing methods and wavelet methods, etc. for profile characterization [2,17]. Next, monitoring of profiles is interpreted as the monitoring of features resulting from related approaches such as projection coefficients or residuals. The majority of the present methods for profile monitoring are exclusively enforceable to a single profile’s data, regardless of their significance. Nonetheless, quality of product is defined as profile data gathered from several channels for certain industrial uses.

Separate monitoring of profile data in each channel distinctively via approaches might be devoid of effectiveness due to the existence of natural interdependence across multichannel profiles. Taking into account cross-correlations between multi-channel profiles, expectations may entail the monitoring of profiles to gain more sensitivity to various shifts. As for studies with focus on the overall multi-channel profiles, [18] integrated multi-channel profiles into an individual profile through summing them up. An alternative approach would be to mix multichannel profiles as a high-dimensional vector followed by the application of methods for dimension reduction, including Principal Components Analysis (PCA) so as to extract characteristics and establish monitoring statistics. Vectorized Principal Components Analysis (VPCA) is also utilized. Conversely, in addition to VPCA violating the structure of correlation in the original data, it mislays certain beneficial demonstrations that are attainable in the original arrangement. Furthermore, as it overlooks functional profiles’ smoothness nature which lessens its effective in cases where the number of sampling points or profiles is large, it can be said to suffer from “the curse of dimensionality” as well. In a more recent attempt, [19] employed a method for projection which is abbreviated as MFPCA (Multichannel Functional Principal Components Analysis); this was done for extracting informative characteristics from multichannel profiles and incorporating the mentioned characteristics to a change-point estimation method intended for analyzing Phase-I.

A few numbers of inquiries within the literature have been carried out on the monitoring of multiple profiles at the same time. [8] introduced a MFPCA approach to extract key features for monitoring image data. [20] developed a robust multivariate control scheme tailored for multivariate functional data, ensuring robustness against both functional anomalies and outliers. [21] presented an innovative functional state-space approach for multi-channel autoregressive profiles, employing the expectation-maximization algorithm and Kalman filter for this purpose. [22] devised a functional monitoring scheme for real-time data, aimed at assessing the temporal stability of fully observed functional quality characteristics. [23] proposed a novel monitoring technique based on uncorrelated multilinear discriminant analysis, which efficiently models multi-channel data to obtain superior monitoring and fault diagnosis performance relative to other approaches. [24] introduced a multi-channel data process monitoring approach in the context of a forging process, utilizing sensor fusion. They proposed an enhanced multilinear feature extraction method, coupled with a feature selection strategy, to improve the separability of profile data. The exploited features are then incorporated with multivariate control charts to monitor multi-channel data. [25] proposed a hierarchical sparse functional principal components analysis (HSMFPCA) method for multistage multivariate profile data. [26] proposed a statistical monitoring scheme for image data by applying a multichannel functional principal components analysis (MFPCA) which extract principal features. The extracted features are used in an EWMA control chart for monitoring the process and detect shifts and change point. [23] proposed a fault detection method and a control chart for multichannel profile data by extracting the important features and incorporating with multivariate control charts. The suggested scheme detects changes in the process effectively. [5] utilized an uncorrelated multilinear principal components analysis for monitoring a nonlinear multichannel profile. [27] suggested a monitoring approach for multichannel profiles in Phase II by applying MFPCA approach and an EWMA control chart. The Markov chain method is incorporated for calculating control limits. [28] used PCA-based approaches for monitoring multichannel profile data and applied to an emission control system. They analyzed the results obtained from two proposed extensions of PCA and compared with traditional PCA exploit to vectors by unfolding the multichannel data. [29] applied a new Sparse Multi- channel Functional Principal Components Analysis (SMFPCA) for modeling multichannel profiles which are correlated weakly. [30] constructed a monitoring method for multivariate sensor-based profiles by considering multichannel functional principal components analysis (MFPCA) and top-R strategy. [31] monitored a multichannel profile in Phase I by incorporating a threshold multivariate principal components analysis. [32] introduced Multilinear principal components analysis (MPCA) to monitor the manufacture process of cylindrical surfaces. Dimension-reduction methods are often initially utilized in order to decrease data dimensionality for nonlinear profiles, given the fact that signals are described as being frequently high-dimensional. A multichannel profile monitoring method was suggested by [33] via principal curves. Moreover, [34] introduced the application of UMPCA to extract features and detect faults and errors. A comparison was conducted among several methods based on PCA by [35] which included MPCA and VPCA for monitoring. [36] recently pushed the application of analyzing functional data further, aimed at profile monitoring; they also established a MFPCA-based change-point model. All in all, the entire said methods usually involve the assumption based on which robust cross-correlations with similar characteristics exist within multichannel profiles and these profiles cannot deal with profiles with varied characteristics effectively; it is due to the fact that various features are integrated together via their extracted PCA loadings. As a result, the monitoring performances of these methods will face serious deterioration.

In recent decades, various control schemes have been developed to monitor changes in the covariance matrix based on the sample covariance matrix *S*. These approaches include utilizing the determinant of *S*, the trace of *S*, and the generalized likelihood ratio (GLR) (refer to [37–39]). [40] developed an enhanced version of the generalized T^2^ chart, utilizing random matrix theory for Phase II monitoring of high-dimensional process means. [41] combined a divide-and-conquer strategy with the MEWMA statistic for monitoring high-dimensional processes where the normality assumption of quality characteristics is relaxed. [42] suggested an integrated monitoring and diagnosis method based on principal components analysis (PCA) for high-dimensional data streams. The use of traditional control charts to monitor the variability of high-dimensional processes shows challenges because the sample covariance matrix is often not positive semi-definite, and its determinant approaches zero. To address this issue, [43] introduced a novel algorithm employing parallelized Monte-Carlo simulation to enhance the sensitivity of two memory-type control schemes for monitoring high-dimensional process variability. They applied various techniques to reduce computational space and runtime. [44] proposed an adaptive LASSO-thresholding-based control chart for Phase II monitoring of multivariate normal quality characteristics in high-dimensional settings and demonstrated the superiority of their control chart over existing ones using different out-of-control patterns. In contrast, [45] concentrated on detecting general shifts in covariance matrix elements without assuming sparsity during Phase II monitoring of high-dimensional process variability. [46] also extended the LASSO-thresholding-based control chart for Phase I monitoring of multivariate processes when the process dimension exceeds the sample size, validating their approach using real industrial data from spur gear production. [47] introduced an approach for tracking shifts in sparse leading eigenvalues between two covariance matrices, studying Phase I monitoring of high-dimensional process variability. [48] integrated ridge penalized likelihood ratio statistics with a multiple dependent state sampling method to monitor high-dimensional covariance. They subsequently presented an improved version of the monitoring method based on generalized multiple dependent state sampling. [49] suggested a monitoring scheme for process variability of high-dimensional data by considering gauge imprecision effect. [50] proposed an adaptive LASSO variability chart for monitoring Phase II of high-dimensional data. [51] introduced a novel approach called the Partitioning Ensemble Control Chart (PECC). The proposed PECC integrates machine learning, ensemble learning, and image partitioning techniques to improve fault detection performance in image-based quality monitoring. The authors developed systematic design and parameter-tuning procedures aimed at optimizing performance in Phase II SPC applications. Through extensive simulation studies—including varying fault sizes and locations—the superiority of PECC over conventional image monitoring schemes was demonstrated. Furthermore, a real-world case study involving an MVS for O-ring quality inspection illustrated the practical applicability and effectiveness of the proposed method. To provide a comprehensive overview of advancements high dimensional process monitoring, [52] conducted a systematic content analysis and conceptual classification of studies related to high-dimensional process monitoring over the period 2004–2024. Based on the review of 72 selected papers, their study identified key research gaps, such as the limited integration of intelligent algorithms, lack of robust real-time applications, and insufficient interpretability of monitoring models. Moreover, they proposed several future research directions, emphasizing the potential of hybrid statistical–machine learning frameworks, adaptive control schemes, and data-driven dimensionality reduction to improve the efficiency and interpretability of control charts in high-dimensional environments. [53] proposed a series of innovative control charts tailored to improve mean shift detection in both high- and low-dimensional processes. Specifically, the authors introduced three novel schemes based on the Srivastava–Du (SD), Bai–Saranadasa (BS), and Dempster (DS) statistics. Through extensive simulation studies and real-world data applications, these charts were evaluated across various multivariate normal and non-normal distributions. The comparative results revealed that the DS and BS charts exhibited similar performance, with the DS chart showing superior detection capability in low-dimensional normal data. Conversely, the SD chart demonstrated enhanced robustness and sensitivity under high-dimensional non-normal conditions. [54] proposed a novel nonparametric Exponentially Weighted Moving Average (EWMA) control scheme that integrates the Random Forest (RF) algorithm with the log-likelihood transformation technique. This hybrid approach converts high-dimensional data into a one-dimensional monitoring statistic, which serves as an input for the EWMA control structure. Simulation experiments demonstrated that the proposed method outperforms existing control charts across various data distributions and process conditions—particularly in detecting single out-of-control (OC) clusters within data streams. To further validate its robustness and practical utility, [54] extended their methodology to real-world case studies, including disk performance monitoring and breast cancer data analysis, confirming the method’s adaptability and superior detection capability in complex monitoring environments.

As far as we know, most of the papers in the area of multi-channel profile monitoring concentrate on proposing a control scheme for change detection in mean of the process, while monitoring variability of the process is important as well as monitoring mean of the process. Investigating Phase II in process monitoring is critical because it directly assesses the stability, and ongoing performance of a process after its initial setup in Phase I. By prioritizing Phase II investigation, organizations can maintain high standards of quality, minimize risks, and ensure long-term operational success. Due to the importance of process variability monitoring, an enhanced Phase-II covariance matrix monitoring framework is proposed for multichannel profiles. This method employs MFPCA to extract a set of features, which efficiently characterize process variations, which are subsequently utilized to develop an EWMA chart. The efficacy of the suggested approach is demonstrated by simulation studies and numerical example. The paper is organized as: in Section 2, the description of MFPCA is explained. Section 3 presented the proposed scheme for monitoring multichannel data. The efficiency of suggested method is assessed in Section 4. A numerical example is conducted in Section 5 to demonstrate the performance of proposed scheme in detecting shifts and the practical implementation of the proposed method is demonstrated through a real case in Section 6. Gradually, some concluding notifications and proposal for further researches are given in Section 7.

## 2. Description of MFPCA

In this Section the description of MFPCA method is reviewed, where it is used for transforming functional data with infinite dimensions in the nature to few features which can show the principal structure of the profiles. Assume that the *p*-channel profile is shaped as $𝐗(u)=\{X_{1}(u),...,X_{p}(u\}$ which forms as Equation (1):


𝐗(u)=μ(u)+𝐘(u), (1)

Where the mean function and the stochastic error are denoted by μ(u) and 𝐘(u), respectively. Moreover, E[𝐘(u)]=0 and u stands for time or space index, typically. let u∈T=[0,1] without loss of generality. 𝐗(u) has infinite dimensions as a profile, which make the monitoring process difficult. Thus, an approach for dimension reduction and extracting features should be applied for process monitoring. The MFPCA is designed for this purpose in this paper.

The strategy of MFPCA is as traditional PCA based on the variability. In other words, MFPCA is based on the covariance of 𝐘(u) which is a function while the traditional PCA is based on the covariance matrix. Assume that $c(u,u^{\prime })=E\{\left\langle 𝐘(u),𝐘(u^{\prime })\right\rangle \}$ as “overall covariance” function of vector-valued random function 𝐘(.) for $u,u^{\prime }\in T$, as an outspoken explanation of the inner product between multidimensional functions; another way to say, $\left\langle f,g\right\rangle =\sum_{j=1}^{p}f_{j}g_{j}$, proposed by [55]. Afterward, the spectral decomposition of $c(u,u^{\prime })$ is as Equation (2):


$$c(u,u^{\prime })=\sum_{k=1}^{\infty }\lambda_{k}v_{k}(u)v_{k}(u^{\prime }).$$ (2)

$\lambda_{k}$ and $v_{k}(u)$ are denoted as eigenvalues and eigenfunctions, which satisfy $\int_{0}^{1}c(u,u^{\prime })v_{k}(u^{\prime })du^{\prime }=\lambda_{k}v_{k}(u),k=1,2,...$.

𝐘(u), Which is an integrable random function is represented based on this eigen decomposition, with the set of eigenfunctions as $𝐘(u)=\sum_{1\leq k<\infty }\xi_{k}v_{k}(u)$, where $\xi_{k}$ is an i.i.d p-variate random variable with mean zero and covariance matrix $\sum_{k}$ and calculates as $\xi_{k}=\int_{0}^{1}𝐘(u)v_{k}(u),k=1,2,...$. We can simply represent that $\sigma_{kj}^{2}=E[\xi_{kj}^{2}]$, where $\xi_{kj}$ is the *j*th component of $\xi_{k}$, and $\lambda_{k}=\sum_{j=1}^{p}\sigma_{kj}^{2}$. Bear in mind, practically, we need to capture some of the eigenvalues and eigenfunctions which provided the substantial variations of multichannel profile.

The description of MFPCA, which considered above supposes that all functions from *p* channels contribute a prevalent set of eigenfunctions and that their interdependence is crucially characterized with correlations among components of $\xi_{k}$. Specifically, MFPCA decreases to the extensively used FPCA when *p = *1, as proposed in [56], in which $\xi_{k}$ is a random variable with mean zero and variance $\lambda_{k}$. Consider Phase II modeling of multichannel profiles. It can be supposed that the observations are gathered due to Equation (3),


$${𝐗}_{i}(u),i=1,2,...=\{\begin{array}{c} {}*20c\mu (u)+{𝐘}_{i}(u)\begin{array}{ccc} {}*20c & & \end{array},i=1,...,\tau ,\\ \mu (u)+\delta (u)+{𝐘}_{i}(u),i=\tau +1,...,m, \end{array}$$ (3)

where τ and μ(u) are denoted the unknown change point and the IC mean function of profiles. Detecting changes in the variability of the process is focused in this paper, as soon as possible. According to the literature of Phase II monitoring, it is assumed that mean function, eigenfunction and covariance matrix (μ(u), $v_{k}(u)$ and $\sum_{k}$) are known. In other words, the IC data for Phase I is adequate for estimating the model perfectly. It is supposed that the IC mean function is equal to zero μ(u)=0 otherwise multichannel profile model (${𝐗}_{i}(u)$) should be transformed to ${𝐗}_{i}(u)-\mu (u)$ for making validity of the assumption. In view of MFPCA, for ${𝐗}_{i}(u),i=1,2,...$ online observations, dimension reduction can be applied by extracting the informative features and projecting on the principal functions. PC-Score (the projections interrelated to the largest d eigenvalues are attained by Equation (4),


$$\xi_{ik}=\int_{0}^{1}{𝐗}_{i}(u)\nu_{k}(u)du,k=1,...,d.$$ (4)

**Fig 1:**
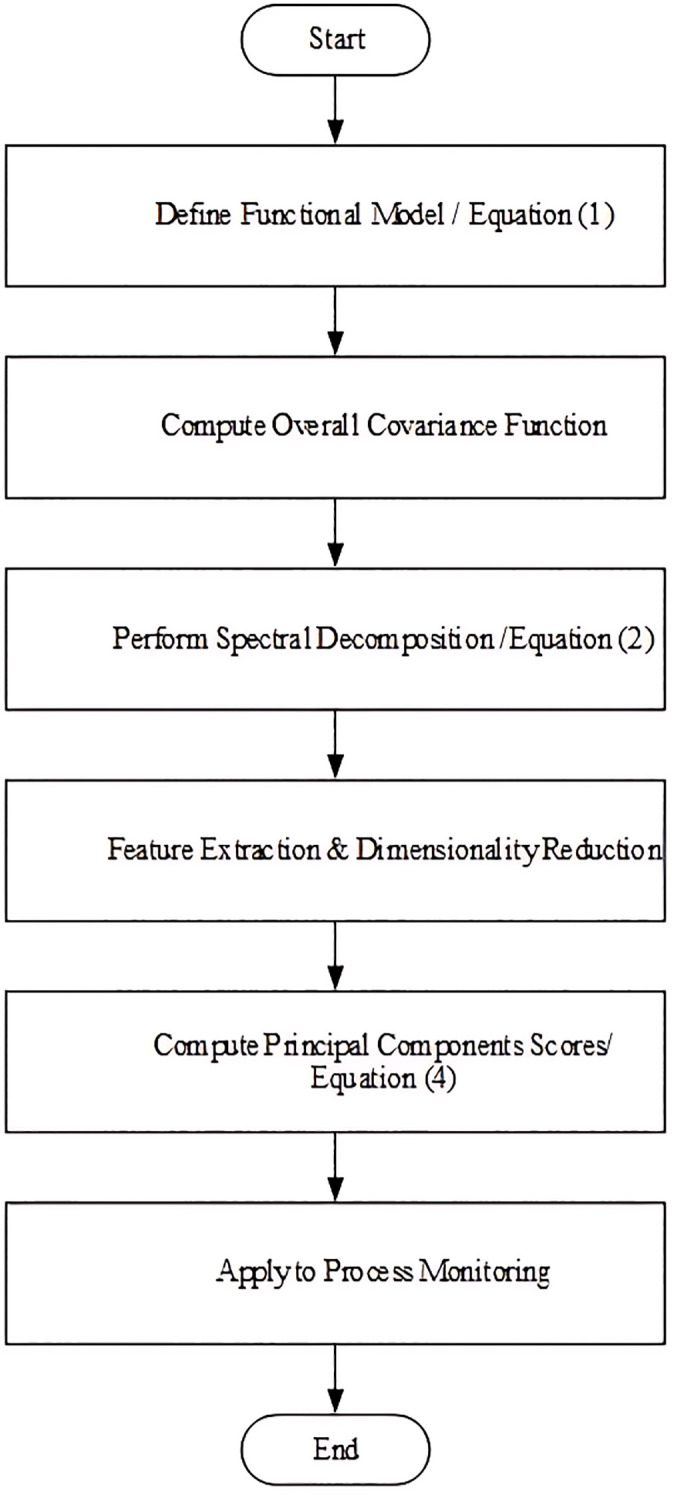
Flowchart of computing MFPCA in Phase II.

Fig 1 represents the process of computing MFPCA and PC-score. The pseudocodes for data generation and computing MFPCA are presented in Appendices A and B, respectively to help the researchers and practitioners to regenerate the results and apply them in real practice applications.

## 3. Proposed scheme for variability monitoring

A monitoring scheme is proposed by constructing an EWMA control chart with the obtained features from MFPCA components. Practically, changes in the mean of the process affect the correlation among the channels even if $\sum_{k}$ remains at its IC value. To detect the modifications in the covariance matrix, we can develop the multivariate exponentially weighted moving covariance matrix suggested by [57] to monitor the PC-scores $\xi_{ik}$ and to build the statistic. When the process is IC, the PC-scores are random vectors with mean 0 and covariance matrix $\sum_{k}$. Consequently, we can construct an EWMA statistic on the basis of $\xi_{ik}$ as;


$${𝐒}_{ik}=(1-\lambda ){𝐒}_{i-1,k}+\lambda \xi_{ik}\xi_{ik}^{T},\;\;\;k=1,2,...,d,$$ (5)

where ${𝐒}_{0k}=\sum_{k}$. For IC situation, $E({𝐒}_{ik})=\sum_{k}$. In this paper, the likelihood ratio statistic is employed to assess the constancy of the process by statistical measures.


$$F_{i}=\sum_{k=1}^{d}\{tr({𝐒}_{ik})-\log|\;{𝐒}_{ik}|-p\}.$$ (6)

**Fig 2:**
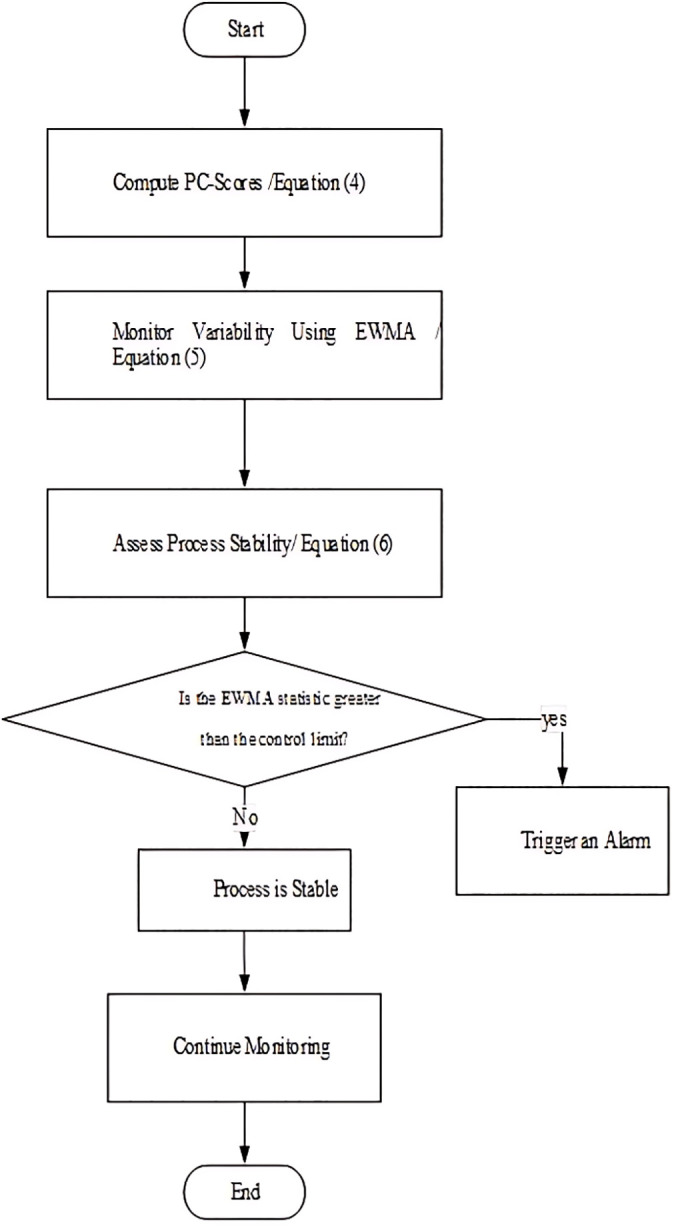
Flowchart of computing the PCWMS statistic in Phase II.

The suggested scheme for the covariance matrix initiates an alert when $F_{i}>L$, where the control limit L is selected to obtain a predetermined IC Average Run Length (ARL). The proposed control chart is called PCWMS. It is noteworthy that determining the correct value of L poses a challenge due to the fact that the statistics $F_{i}$ lack a recognized and standardized distribution. Typically, we must derive L through simulations based on specified design parameters like λ, and IC ARL. Fig 2 represents the process of computing the PCWMS statistic. Also, the pseudocode for computing the PCWMS statistic is presented in Appendix C.

## 4. Simulation studies

The efficiency of suggested scheme is appraised in detecting the shift of multichannel profile via ARL and SDRL comparisons. Average Run Length (ARL) and Standard Deviation of Run Length (SDRL) provide crucial insights into the performance of control charts. ARL is directly linked to the system’s sensitivity in detecting deviations or shifts from the norm. A shorter ARL indicates that the system will detect issues quickly, which is crucial for early intervention, especially in high-stakes environments like manufacturing, or healthcare systems. While ARL measures the average points until getting a signal from a control chart, SDRL tells you how reliable and stable that detection process is. In many simulations, especially in process control or manufacturing, it is not just about detecting issues early but doing so in a consistent, predictable manner. High SDRL can indicate that sometimes the system responds very quickly, but at other times, it might take much longer to detect issues, which can lead to erratic behavior and inefficiencies. A large SDRL can also indicate that the system either is prone to excessive false alarms or sometimes misses early detection.

**Fig 3:**
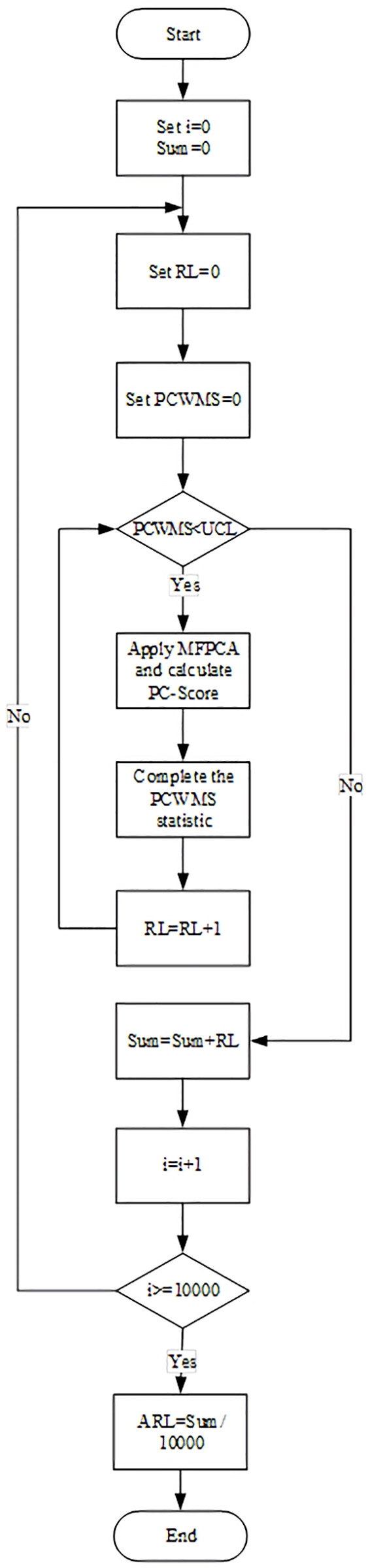
Flowchart of computing ARL for the PCWMS control chart in Phase II.

For simpleness and stability with relevant literature, it is supposed to consider change point equal to 0 for OC scenarios and IC ARL = 200. The numerical results are assessed with 10000 replications in this paper. The channels number is equal to   p=4. According to the generic IC model ${𝐗}_{i}(u)=\mu (u)+{𝐘}_{i}(u),$ that u∈[0,1], the in-control mean functions are zero. The processes are sampled on a grid of n=50 equidistant points in T=[0,1]. Fig 3 represents the process of computing ARL in the proposed control chart. Moreover, the pseudocode for computing the ARL and SDRL of the proposed control chart is presented in Appendix D.

We consider ${𝐘}_{i}(u)=\sum_{k=1}^{d}\xi_{ik}\upsilon_{k}(u),$ where $\upsilon_{k}(u)$ are the first four non-constant and Fourier basis functions with a base period of 0.5, and $\xi_{ik}$ (*p*-dimensional) follows a multivariate normally distribution with mean vector **0** and covariance matrix $(\sum_{k}{)}_{ij}=k(\rho {)}^{\left|i-j\right|}$. The correlation is considered as ρ=0.8. The dimension of the covariance matrix $\sum_{k}$ in the multichannel profile relates to the interrelationship among the p channels.

The quantity and diversity of out-of-control models is large enough to let a universal and pervasive comparison. Only three delegate scenarios for shift are considered, where *δ* ∈{0,0.1,0.2,0.3,0.5,0.75,1} is the shift size. The proposed out-of-control scenarios are as follows:

The efficiency of the suggested control scheme under three OC scenarios is assessed in terms of ARL and SDRL, in this section. Type of shifts consist of diagonal, off-diagonal and synchronous diagonal/off-diagonal disturbances as follows:

**Scenario 1:** The occurrence of assignable cause leads to shifts in all covariance matrix elements.
**Scenario 2:** The occurrence of assignable cause affects all variance elements while the covariance elements remain unchanged.
**Scenario 3:** The OC covariance matrix includes off-diagonal disturbances

**Fig 4:**
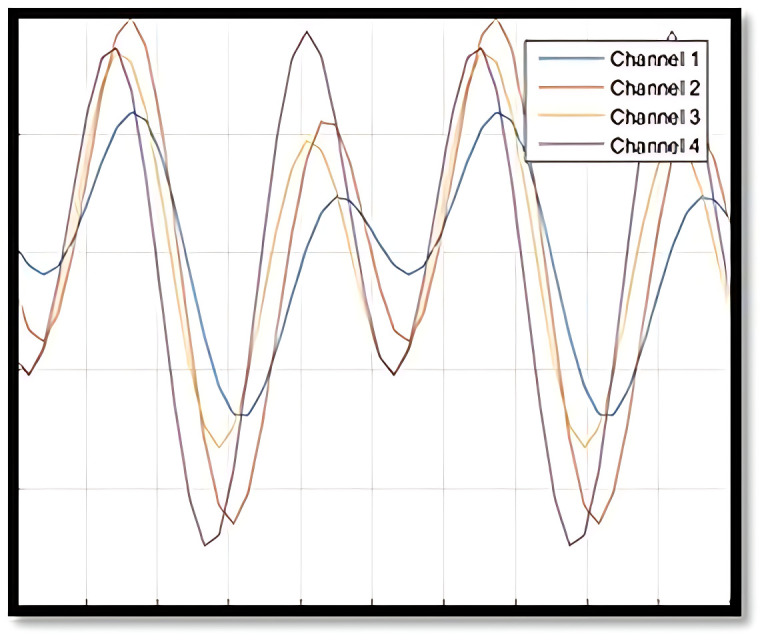
The illustration of IC model.

**Fig 5:**
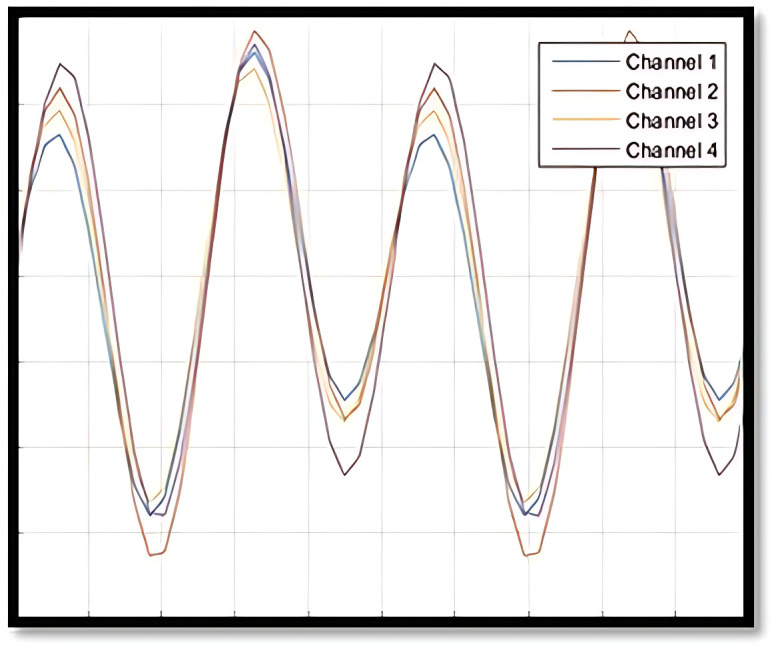
The illustration of OC model- scenario 1.

**Fig 6:**
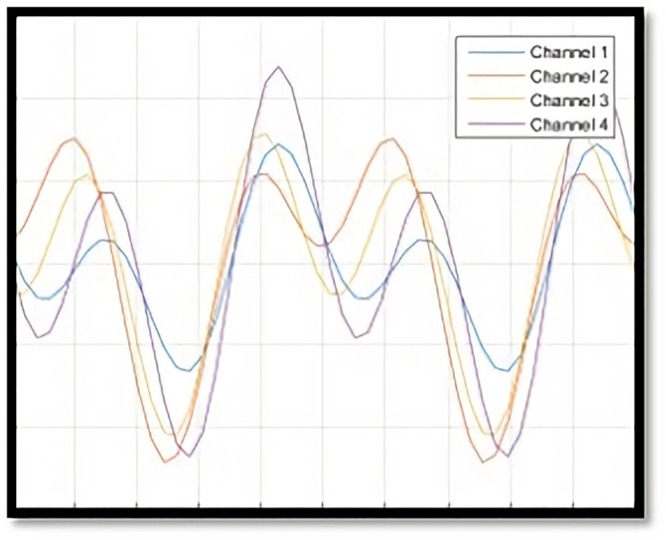
The illustration of OC model- scenario 2.

**Fig 7:**
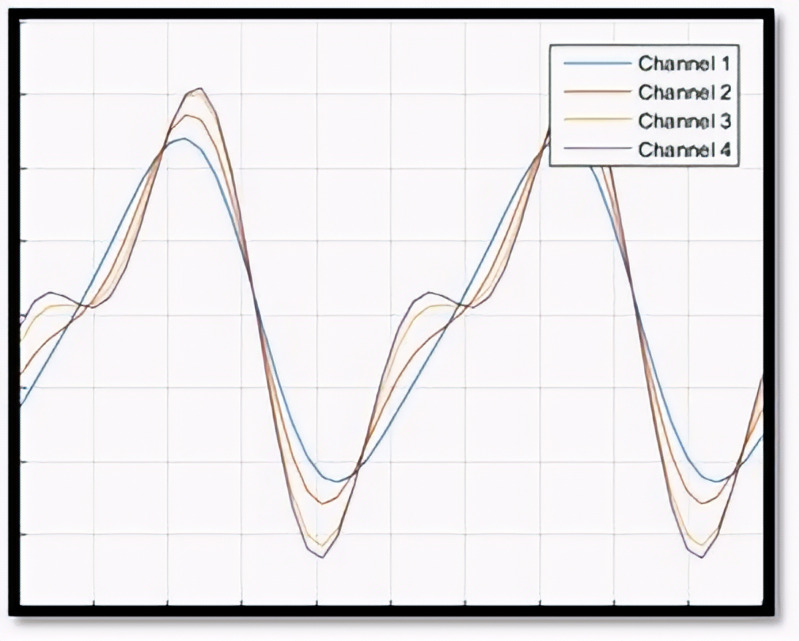
The illustration of OC model- scenario 3.

Given the allowance for various IC and OC shapes, multiple scenarios can arise in an OC situation during Phase II. To provide a clearer understanding of OC definitions and the similarities or differences in OC shapes within Phase II profile monitoring, Figs 4–7 illustrate several IC and OC profiles related to the multi-channel profile. Fig 4 shows an IC model for multi-channel profile that has 4 channels. Fig 5 illustrates an OC model in scenario 1, under shifts in all covariance matrix elements. Fig 6 depicts an OC model in scenario 2, under shifts in all variance elements while the covariance elements remain unchanged. Fig 7 shows an OC model in scenario 3, which includes off-diagonal disturbances in the covariance matrix.

Simulation studies report ARLs and Standard Deviation of run Length (SDRLs) of the proposed control chart under the defined OC scenarios when *δ* ∈{0,0.1,0.2,0.3,0.5,0.75,1} and the results are reported in Tables 1–10. The shift size (δ) in the covariance matrix was tested from 0 to 1 with various increments to systematically evaluate how different magnitudes of process shifts affect the performance of the proposed control chart. Small shifts (δ close to 0) represent minor process variations that are harder to detect, testing the ability of the control chart to identify subtle deviations. Larger shifts (δ approaching 1) simulate significant process changes, helping to evaluate how quickly and effectively the monitoring system responds. Evaluating different δ values ensures that the model is not overly sensitive to minor fluctuations (reducing false alarms) while still being responsive to meaningful process changes.

**Table 1:** Values of OC ARL and SDRL with different shifts when n = 10, 50, 100 under Scenario1.

*δ*	Criterion	80%			99%		
		*n* = 10	*n* = 50	*n* = 100	*n* = 10	*n* = 50	*n* = 100
0	ARL	200.012	200.012	200.012	200.012	200.012	200.012
	SDRL	201.306	199.722	202.892	202.215	199.367	203.351
0.1	ARL	84.916	82.128	80.216	86.011	84.256	82.325
	SDRL	83.256	81.653	74.652	85.796	83.362	75.695
0.2	ARL	25.442	21.238	20.823	26.289	23.614	21.082
	SDRL	23.256	20.975	16.785	25.369	22.154	20.145
0.3	ARL	13.309	11.264	10.031	14.058	12.658	11.869
	SDRL	12.085	10.264	7.25	12.857	11.378	8.654
0.5	ARL	7.087	5.421	4.848	7.628	6.985	4.971
	SDRL	6.345	4.125	3.452	6.854	7.346	4.297
0.75	ARL	3.178	2.317	1.976	3.692	2.749	2.038
	SDRL	2.409	1.682	1627	2.584	1.635	1.895
1	ARL	1.909	1.826	1.403	2.031	1.965	1.832
	SDRL	0.956	0.903	0.924	0.993	0.873	1.125

**Table 10:** ARLs and SDRLs for the proposed control chart by VFPCA and MFPCA approaches with different correlations.

*δ*	Criterion	high-level correlation (*ρ = 0.8*)		low-level correlation (*ρ = 0.4*)	
		VFPCA	MFPCA	VFPCA	MFPCA
0	ARL	200.196	200.012	201.454	202.308
	SDRL	199.894	201.951	202.89	201.432
0.1	ARL	172.084	159.345	168.180	152.407
	SDRL	170.258	158.723	167.227	151.254
0.2	ARL	97.119	90.116	99.548	91.802
	SDRL	96.314	89.637	98.213	90.036
0.3	ARL	53.609	44.135	57.314	41.324
	SDRL	52.176	43.004	55.381	40.118
0.5	ARL	18.358	12.815	21.199	9.036
	SDRL	17.980	12.305	20.037	8.884
0.75	ARL	6.214	4.961	8.117	4.182
	SDRL	5.721	4.108	7.922	3.695
1	ARL	3.687	2.706	4.528	1.934
	SDRL	2.984	2.071	4.024	1.683

**Table 2:** Values of OC ARL and SDRL with different shifts when n = 10, 50, 100 under Scenario2.

*δ*	Criterion	80%			99%		
		*n* = 10	*n* = 50	*n* = 100	*n* = 10	*n* = 50	*n* = 100
0	ARL	200.012	200.012	200.012	200.012	200.012	200.012
	SDRL	199.347	198.415	198.756	202.956	198.367	201.652
0.1	ARL	189.814	186.102	185.938	190.018	189.352	188.266
	SDRL	188.524	184.667	185.720	188.703	188.361	186.378
0.2	ARL	121.546	119.361	118.672	123.098	122.374	120.413
	SDRL	120.225	120.653	112.106	121.635	121.875	117.118
0.3	ARL	69.064	66.506	65.023	71.241	68.105	66.850
	SDRL	67.924	64.317	63.249	71.625	67.095	61.029
0.5	ARL	18.315	15.019	12.814	19.237	16.369	14.068
	SDRL	17.165	13.758	12.280	18.452	15.274	13.883
0.75	ARL	4.216	3.421	2.547	5.194	4.082	3.515
	SDRL	3.769	3.652	2.131	4.361	3.642	2.433
1	ARL	1.942	1.874	1.623	2.146	1.937	1.849
	SDRL	0.963	0.875	0.734	1.036	0.892	0.855

**Table 3:** Values of OC ARL and SDRL with different shifts when n = 10, 50, 100 under Scenario3.

*δ*	Criterion	80%			99%		
		*n* = 10	*n* = 50	*n* = 100	*n* = 10	*n* = 50	*n* = 100
0	ARL	200.012	200.012	200.012	200.012	200.012	200.012
	SDRL	201.985	202.892	199.267	198.361	201.378	200.608
0.1	ARL	169.916	164.695	162.903	173.625	171.328	170.004
	SDRL	168.267	160.002	159.810	172.163	170.039	169.256
0.2	ARL	99.335	93.499	93.314	101.698	99.892	99.128
	SDRL	98.337	91.284	91.207	100.241	98.203	96.416
0.3	ARL	53.218	49.153	48.462	56.019	55.364	54.032
	SDRL	51.893	46.579	45.953	55.106	54.114	53.128
0.5	ARL	18.314	16.879	16.263	21.964	19.385	18.329
	SDRL	16.885	15.485	14.740	20.569	17.901	17.358
0.75	ARL	9.129	6.912	6.011	10.211	9.358	8.167
	SDRL	8.452	5.752	5.116	9.308	8.637	8.063
1	ARL	4.027	3.118	2.891	6.917	6.385	5.244
	SDRL	2.857	2.985	2.659	5.496	5.874	5.129

**Table 4:** Values of OC ARL and SDRL with different shifts based on different percentages of total variation, under Scenario1.

*δ*	Criterion	75%	80%	85%	90%	95%	99%
0	ARL	200.012	200.012	200.012	200.012	200.012	200.012
	SDRL	198.378	202.892	198.575	199.364	201.776	203.351
0.1	ARL	70.134	75.216	74.325	74.628	76.248	78.325
	SDRL	69.206	74.652	73.625	73.139	75.163	75.695
0.2	ARL	15.679	17.823	16.468	16.025	17.659	21.082
	SDRL	14.362	16.785	15.247	15.823	16.745	20.145
0.3	ARL	7.803	8.031	8.126	7.936	8.264	9.869
	SDRL	6.526	7.25	7.952	7.026	7.541	8.654
0.5	ARL	3.218	3.848	3.105	3.268	3.925	4.971
	SDRL	3.316	3.452	2.167	2.567	3.026	4.297
0.75	ARL	1.730	1.976	1.863	1.903	2.005	2.038
	SDRL	1.429	1627	1.196	1.253	1.364	1.895
1	ARL	1.316	1.403	1.236	1.310	1.724	1.832
	SDRL	0.985	0.924	0.841	0.875	1.003	1.125

**Table 5:** Values of OC ARL and SDRL with different shifts based on different percentages of total variation, under Scenario2.

δ	Criterion	75%	80%	85%	90%	95%	99%
0	ARL	200.012	200.012	200.012	200.012	200.012	200.012
	SDRL	199.326	198.756	201.745	202.985	203.174	201.652
0.1	ARL	180.769	182.938	178.634	180.937	185.052	188.266
	SDRL	184.675	185.720	177.374	179.587	183.957	186.378
0.2	ARL	110.362	113.672	107.928	111.075	116.598	120.413
	SDRL	111.126	112.106	106.410	110.475	115.197	117.118
0.3	ARL	59.795	62.023	58.029	60.134	61.322	63.850
	SDRL	58.362	61.249	57.143	58.995	60.109	61.029
0.5	ARL	11.682	12.814	11.308	11.857	12.961	14.068
	SDRL	10.876	12.280	10.25	10.417	11.399	13.883
0.75	ARL	2.107	2.547	2.361	2.676	2.983	3.515
	SDRL	1.985	2.131	1.896	2.179	2.394	2.433
1	ARL	1.352	1.623	1.421	1.463	1.698	1.849
	SDRL	0.617	0.734	0.752	0.685	0.968	0.855

**Table 6:** Values of OC ARL and SDRL with different shifts based on different percentages of total variation, under Scenario3.

δ	Criterion	75%	80%	85%	90%	95%	99%
0	ARL	200.012	200.012	200.012	200.012	200.012	200.012
	SDRL	198.376	202.892	201.951	202.385	201.985	203.608
0.1	ARL	160.326	162.695	159.345	161.225	167.956	170.004
	SDRL	158.364	159.002	158.723	160.836	166.206	169.256
0.2	ARL	91.854	93.499	90.116	92.851	96.124	99.128
	SDRL	90.231	91.284	89.637	91.653	95.776	96.416
0.3	ARL	44.064	48.153	44.135	48.298	50.208	54.032
	SDRL	43.258	46.579	43.004	46.993	49.871	53.128
0.5	ARL	14.166	15.879	12.815	15.457	16.006	18.329
	SDRL	13.125	15.485	12.305	14.870	15.271	18.358
0.75	ARL	5.204	6.912	4.961	5.652	7.344	8.167
	SDRL	4.985	6.752	4.108	4.937	6.732	8.063
1	ARL	2.895	3.118	2.706	3.264	4.128	5.244
	SDRL	2.764	2.985	2.071	2.841	3.842	5.129

**Table 7:** Values of OC ARL and SDRL with different smoothing parameters under Scenario1.

*δ*	Criterion	λ=\bold0.\bold2			λ=\bold0.\bold1			λ=\bold0.\bold0\bold5		
		75%	80%	99%	75%	80%	99%	75%	80%	99%
0	ARL	200.012	200.012	200.012	200.012	200.012	200.012	200.012	200.012	200.012
	SDRL	198.378	202.892	203.351	199.145	202.069	202.552	201.365	201.147	199.245
0.1	ARL	70.134	75.216	78.325	69.076	73.364	75.815	68.289	71.627	73.288
	SDRL	69.206	74.652	75.695	68.023	7.258	74.221	67.314	71.208	72.985
0.2	ARL	15.679	17.823	21.082	14.361	15.035	19.486	14.268	14.801	17.621
	SDRL	14.362	16.785	20.145	13.205	14.212	18.259	14.036	14.031	16.415
0.3	ARL	7.803	8.031	9.869	6.132	7.872	8.267	5.994	7.026	7.930
	SDRL	6.526	7.25	8.654	5.785	7.196	7.965	5.146	6.541	7.264
0.5	ARL	2.781	3.095	3.645	2.907	3.269	4.165	3.218	3.848	4.971
	SDRL	2.178	2.486	3.098	2.541	2.953	3.894	3.316	3.452	4.297
0.75	ARL	1.398	1.627	1.880	1.519	1.831	1.983	1.730	1.976	2.038
	SDRL	1.007	1.229	1.317	1.241	1.340	1.409	1.429	1.618	1.895
1	ARL	1.109	1.158	1.376	1.299	1.272	1.620	1.316	1.403	1.832
	SDRL	0.658	0.853	0.974	0.814	0.785	1.031	0.985	0.924	1.125

**Table 8:** Values of OC ARL and SDRL with different smoothing parameters under Scenario 2.

*δ*	Criterion	λ=\bold0.\bold2			λ=\bold0.\bold1			λ=\bold0.\bold05		
		75%	80%	99%	75%	80%	99%	75%	80%	99%
0	ARL	200.012	200.012	200.012	200.012	200.012	200.012	200.012	200.012	200.012
	SDRL	199.326	198.756	201.652	200.146	199.037	201.895	203.827	200.365	198.245
0.1	ARL	180.769	182.938	188.266	179.371	180.791	184.278	175.236	176.523	181.309
	SDRL	184.675	185.720	186.378	170.389	180.684	183.336	170.288	177.125	184.064
0.2	ARL	110.362	113.672	120.413	108.264	111.145	116.296	104.125	108.162	114.028
	SDRL	111.126	112.106	117.118	107.724	110.694	115.285	102.301	108.625	111.237
0.3	ARL	59.795	62.023	63.850	57.034	59.801	61.370	52.069	57.654	60.003
	SDRL	58.362	61.249	61.029	59.247	58.769	60.251	51.369	56.748	61.763
0.5	ARL	8.114	9.615	10.294	10.150	11.265	13.614	11.682	12.814	14.068
	SDRL	8.003	8.661	10.225	9.592	10.347	12.407	10.876	12.280	13.883
0.75	ARL	1.527	1.937	2.308	1.982	2.312	2.982	2.107	2.547	3.515
	SDRL	1.103	1.856	2.187	2.014	2.136	2.208	1.985	2.131	2.433
1	ARL	1.005	1.029	1.357	1.074	1.206	1.563	1.352	1.623	1.849
	SDRL	0.690	0.751	0.876	0.927	0.826	0.892	0.617	0.734	0.855

**Table 9:** Values of OC ARL and SDRL with different smoothing parameters under Scenario 3.

*δ*	Criterion	λ=\bold0.\bold2			λ=\bold0.\bold1			λ=\bold0.\bold05		
		75%	80%	99%	75%	80%	99%	75%	80%	99%
0	ARL	200.012	200.012	200.012	200.012	200.012	200.012	200.012	200.012	200.012
	SDRL	198.376	202.892	203.608	199.143	202.060	198.354	201.369	201.472	200.954
0.1	ARL	160.326	162.695	170.004	153.650	155.925	164.368	149.024	152.312	161.967
	SDRL	158.364	159.002	169.256	151.023	153.875	162.852	147.102	152.118	159.485
0.2	ARL	91.854	93.499	99.128	87.104	90.384	97.861	85.319	87.932	92.028
	SDRL	90.231	91.284	96.416	85.339	88.771	95.357	83.018	86.712	90.632
0.3	ARL	44.064	48.153	54.032	42.187	45.362	51.309	38.210	41.598	47.249
	SDRL	43.258	46.579	53.128	42.036	44.237	50.851	37.529	40.044	45.922
0.5	ARL	11.894	13.269	15.147	13.116	14.059	16.385	14.166	15.879	18.329
	SDRL	11.569	12.824	14.776	12.986	13.997	15.208	13.125	15.485	18.358
0.75	ARL	4.088	5.173	6.362	5.627	6.203	7.196	5.204	6.912	8.167
	SDRL	3.685	4.925	5.881	5.231	5.770	6.958	4.985	6.752	8.063
1	ARL	1.829	2.039	3.870	2.543	2.947	4.035	2.895	3.118	5.244
	SDRL	1.671	1.801	3.726	2.302	2.725	3.722	2.764	2.985	5.129

Practically, each functional profile is only observable in discrete points. The parameter *n* specifies the number of these sampling points on each profile. For Phase I and Phase II, the quantity and locations of the grid points must be carefully selected to accurately depict the functional curve. It is advisable to use identical numbers and positions for Phase I and Phase II samples.

Given that μ(u) is derived using smoothing techniques, the selection of *n* parallels that in nonparametric profiles [1,2]. Typically, higher observation noise or measurement error, or profiles with significant fluctuations or high-frequency changes, necessitate a larger *n* to enhance the signal-to-noise ratio and improve detection accuracy. The Nyquist-Shannon sampling theorem (Shannon 1949) essentially offers guidance on this matter. In the absence of specific information, choosing a larger *n* is generally prudent, despite the increased computational demand. According to simulation outcomes in Section 3, it is recommended that the number *n* of grid points in each multichannel profile must be at least 50.

To demonstrate the effect of n, Tables 1–3 represents the values of ARL and SDRL for different values of n in multichannel profile under the defined scenarios. It obviously represents that the suggested scheme with a larger n, detects the out-of-control alarm faster however the differences of results become insignificant for n>50. Thus, the grid points in the simulation reports of this paper are selected n=50.

According to Tables 1–3, the selection of *n = *50 is justified as it provides an optimal balance between detection speed and stability in monitoring performance. The Average Run Length (ARL) values show that for moderate to large shifts (δ ≥ 0.2), *n = *50 detects out-of-control (OC) conditions significantly faster than *n = 10*, while performing nearly similar to *n = *100. Additionally, the Standard Deviation of Run Length (SDRL) values indicate that *n* = 50 offers more stable detection compared to *n = *10, reducing variability in run length. Although *n = *100 achieves slightly better performance, the improvement is marginal, while the computational cost and data collection efforts increase. Thus, *n* = 50 is chosen as it ensures efficient monitoring with a good trade-off between detection speed, stability, and practicality.

Consider that d is the number of eigenfunctions utilized for projection, or, equivalently, the number of PCs examined. In Phase-II monitoring d is supposed to be fixed and has been estimated accurately in Phase I. There are lots of schemes suggested related to the selection of d. It is hard to choose d by cross-validation or by using pseudo-Akaike information criterion (AIC) because no response has been found in online monitoring. We distinguished d due to the percentage of total variation described by the extracted PC-scores. These percentages are often used because they provide a reasonable trade-off between capturing enough variation in the data and keeping the model simple and computationally manageable. In this case, the variation described by the extracted PC-scores likely refers to the proportion of the total data variation explained by each selected eigen function or PC. This method ensures that a sufficient proportion of the original data’s variability is preserved in the reduced set of PCs, allowing for effective monitoring without the need for cross-validation or response-based criteria [58]. Tables 4–6 represents that this selection hand overs advisedly desirable performance, and the differences among ARL values are insignificant, particularly for percentages between 85 and 95.

Moreover, OC ARL and SDRL values are compared where d is selected to describe the various percentages of total variation in Tables 4–6 according to scenarios 1–3. From Tables 4–6, it can be concluded that when d is elected on the basis of high proportion, the out-of-control ARLs as an alternative, increase more than others. It represents that a larger d does not essentially result in the development of out-of-control efficiency and that selecting an appropriate d is significant. Moreover, these tables present that the efficiencies are entirely steady, particularly for percentages between 85 and 95. Hence, in paper we applied 90 percent of total variation as the criterion to choose d.

According to Tables 4–6, the selection of 90% variation is justified based on the balance it provides between fast detection of out-of-control (OC) conditions and stability in monitoring performance. Looking at the Average Run Length (ARL) values, a 90% variation achieves relatively low ARL values for moderate to large shifts (*δ ≥ *0.2), ensuring quicker detection of process deviations compared to lower percentages like 75% or 80%. Meanwhile, for small shifts (*δ = *0.1), the ARL remains competitive, meaning the system does not respond too quickly to minor variations, which could lead to false alarms. Additionally, the Standard Deviation of Run Length (SDRL) values indicate that 90% variation provides greater stability in run length compared to lower percentages, as it avoids excessive variability in performance. While 99% variation offers slightly lower ARL for larger shifts, it also increases SDRL, making the monitoring process less consistent. Thus, 90% variation is chosen as it ensures efficient, stable, and timely detection of shifts without unnecessary sensitivity to small fluctuations.

The selection of 90% percentage variation over 85% is based on achieving a better trade-off between fast detection, stability, and reliability in monitoring performance. When comparing the ARL values, 85% variation performs similarly to 90% for small to moderate shifts, but 90% has a slight advantage in detecting larger shifts faster. For instance, in Table 4, at (*δ = 0.5*), ARL for 85% is 3.105, whereas for 90% it is 3.268, showing that both variations are close in performance, but 90% may provide a more robust detection.

Additionally, SDRL values indicate that 90% variation results in more stable detection times, as its SDRL values are consistently moderate across different shift sizes. In contrast, 85% variation sometimes shows slightly higher SDRL fluctuations, which could lead to inconsistent monitoring performance. While 85% is a reasonable choice, 90% is slightly preferred as it ensures a balanced response—avoiding excessive false alarms while still detecting shifts efficiently and consistently.

The impact of smoothing parameter λ on the performance of suggested scheme is considered in Tables 7–9. The OC ARL and SDRL of suggested scheme under three scenarios with distinct smoothing parameters λ =0.2, 0.1 and 0.05 are compared. According to the results, the suggested scheme with λ≥0.2 outperforms the other values of λ under all scenarios. Particularly, the proposed control chart by larger λ is better than the ones by smaller λ in detecting large shifts, where the proposed control chart with smaller λ is superior to the ones with larger λ in detecting small shifts.

In simulation reports in this paper, it is supposed that the in-control parameters are known or, likewise, which are estimated from an adequate large reference data set.

One way to monitor multichannel profiles is by stacking the profiles from each channel and converting them into a high-dimensional vector. PCA can then be applied to this vector to extract features for constructing an EWMA control chart. This approach is known as vectorized-PCA EWMA (VPWMS). However, there are several challenges associated with using VPCA for analyzing multichannel profiles. As previously noted, this method disrupts the original correlation structure, leading to a loss of valuable data representations. Additionally, the effectiveness of VPWMS declines as an excessive number of grid points increase the dimensionality and introduce significant stochastic noise. Table 10 displays the ARL and SDRL values obtained using the VFPCA and MFPCA approaches, accounting for different magnitudes of changes in the covariance matrix, with the smoothing parameter set to 0.2. The results show that the suggested control chart (PCWMS) demonstrates greater efficiency under both high and low-level correlations between profiles. Moreover, we can use VFPCA instead of MFPCA and build a VPWMS statistic to compare their results and performance. Additionally, as ρ increases, the detection power of shifts by VPWMS deteriorates. Conversely, the PCWMS scheme achieve superior results with higher *ρ*, as it efficiently incorporates the inter-channel correlation information into the model.

## 5. Illustrative examples and applications

### 5.1 Simplified demonstration example

**Fig 8:**
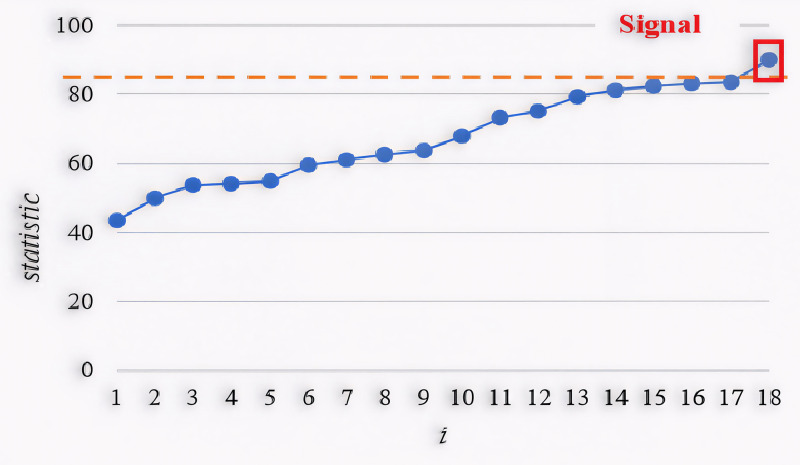
Proposed PCWMS control chart in detecting shift in variability with MFPCA approach.

An illustrative example based on Section 4 data is shown to represent the application of the suggested approach. X-axis represents the sample index, meaning each point corresponds to a new observation in the dataset and the monitoring statistic value, which is used to detect out-of-control (OC) conditions in the covariance structure, is presented in Y-axis. Dashed orange line is the Upper Control Limit (UCL). If the statistic exceeds this limit, an OC signal is triggered, indicating a significant shift in the covariance matrix. The monitoring statistic gradually increases, suggesting that variability in the covariance matrix is accumulating. Out-of-control multi-channel profile data is generated and a shift (δ=0.75, scenario 1) is induced in the covariance matrix till the proposed statistic exceeds the UCL. The proposed chart is plotted and represented in Fig 8. It can be figured out, an OC signal at 18^th^ sample. Note that the suggested control scheme to monitor the variability of covariance matrix in multi-channel profile data is implemented in MATLAB and can be simply utilized by practitioners in real world problems. If an OC signal is detected, practitioners should investigate potential root causes of the shift (e.g., sensor drift, external disturbances). Possible corrective actions include adjusting process parameters, recalibrating sensors, or applying further diagnostic analysis.

### 5.2 Case study: Multi-operation forging process

We demonstrate the practical implementation of the proposed method through a multi-operation forging process, outlining each step in detail. In this process, the forging machine consists of multiple dies, each responsible for executing a specific operation within a single stroke. The forces applied to all dies are monitored using four strain gauge sensors attached to the press’s four columns. During each operational cycle, the system records four-channel tonnage profiles.

A dataset of 220 multichannel profiles was gathered under various experimental conditions, comprising 151 in-control (IC) profiles obtained during normal production and 69 out-of-control (OC) profiles recorded for each sensor when a component was missing at the piercing station (Data and a readme file are available in the supplementary material of the paper). Notably, the profiles associated with piercing faults closely resemble those from normal operations, making fault detection challenging. This case study focuses on developing a Phase-II monitoring method, for which the IC profiles are used separately to estimate the necessary parameters. The implementation steps are outlined below:

**Step 1: Compute the Relevant IC Parameters**

The Multivariate Functional Principal Component Analysis (MFPCA) is applied to $m_{\text{0}}=\text{1}\text{5}\text{1}$ in-control (IC) profile samples to determine the necessary parameters. This process involves calculating the mean function and identifying the first d=16 eigenfunctions, whose associated eigenvalues collectively explain more than 85% of the total variation in the profiles. Since the profiles are recorded at n=200 discrete points, the corresponding covariance matrix is then estimated.

**Step 2: Set Parameters and Determine Control Limit**

Select the desired in-control average run length (IC ARL) and the smoothing constant λ. The control limit *L* is determined based on *p*, *d*, the IC ARL, and λ. Given the parameters p=4, d=16, ${ARL}_{\text{0}}=\text{2}\text{0}\text{0}$, and λ = 0.2, the calculated control limit is *L* = 87.3. Using these values, the PCWMS control chart is then constructed.

**Step 3: Initiate Process Monitoring**

**Fig 9:**
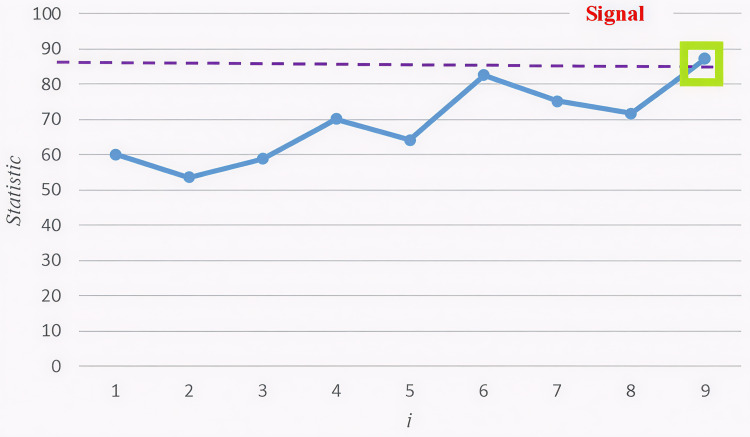
Proposed PCWMS control chart in detecting shift in variability with MFPCA approach.

To validate the effectiveness of the proposed method, we use 69 OC samples of tonnage data. The PCWMS chart promptly identifies the shift at the 9^th^ sample, with$\;F_{i}$ above the control limit. The proposed chart is plotted and represented in Fig 9.

## 6. Conclusions and future directions

Covariance matrix monitoring considering Phase II multichannel profile is an impressive field, which has not been concentrated thoroughly. The PCWMS control chart is based on the combination of MFPCA method and EWMA control chart that consider the correlation of multichannel data. The proposed scheme enables satisfactory performance and is simple to design. From a practical perspective, the proposed control chart can be applied to industries where multichannel data is common, such as manufacturing and healthcare. To probe the efficiency of the suggested control scheme, three OC scenarios including both diagonal and non-diagonal shifts in covariance elements were specified. Sensitivity analysis was carried out by extensive simulation reports in terms of ARL and SDRL for investigating the validity of the proposed control scheme. Gradually, an illustrative example is implemented to depict the shift detection power of the suggested approach. Based on the results, it is recommended that the number *n* of grid points in each multichannel profile must be at least 50. Also, it can be concluded that a larger *d* does not essentially result in the development of out-of-control efficiency and that the selecting a proper *d* is significant. Moreover, results present that the efficiencies are entirely steady, particularly for percentages between 85 and 95. As another consequence of this paper, the proposed control chart by larger λ is superior to the ones by smaller λ in detecting large shifts, where the proposed control chart with smaller λ is better than the ones with larger λ in detecting small shifts.

However, there are certain limitations to the proposed control chart that should be considered. First, the method assumes that data is relatively clean and free from noise, and its performance in the presence of measurement errors or missing data requires further investigation. Furthermore, the current framework may face scalability issues when applied to very large datasets or real-time monitoring systems, where computational resources could become a bottleneck. Additionally, while the study focused on diagonal and non-diagonal shifts in covariance elements, more complex shifts and non-linear patterns could present challenges that warrant further exploration. Looking ahead, several promising areas for future research could improve the applicability of this control chart. Investigating the effect of measurement errors on the proposed control chart could be a promising avenue for potential future research. Moreover, applying other approaches for dimension reductions such as tensor decomposition, developing a diagnostic approach for identifying the change point and designing a joint monitoring scheme for mean and variability of multichannel data are further suggestions for potential future directions. Using machine learning techniques such as Support Vector Machine, Random Forest, Artificial Neural Network, Decision Tree and so on, are strongly recommended to address high dimensionality problem in multichannel profile monitoring. These techniques can be applied instead of control charts or in combination of control charts to address the problems in monitoring high dimensional data (Refer to [59–61]). Another promising direction for future research is to explore the applicability of the proposed approach in other fields that involve complex signal patterns and high-dimensional data. In particular, it can be extended to areas such as EEG-based health data analysis, waveform signal processing, near-infrared (NIR) fluorescence detection, molecular networking, and spectroscopy signal interpretation. For instance, EEG data have been widely used to assess neural connectivity and cognitive states through advanced correlation-based techniques [62]. In the field of mechanical systems, waveform decomposition and variational filtering methods have been successfully applied to extract fault-related features from nonstationary vibration signals [63]. Similarly, NIR fluorescent probes have enabled highly selective detection of specific ions and molecules in biological and environmental systems [64], while molecular networking strategies have facilitated comprehensive characterization of complex molecular compositions [65]. Moreover, NIR spectroscopy signals have proven effective for physiological state classification and monitoring during anesthesia [66]. These studies collectively indicate that the proposed framework has the potential to be generalized and optimized for a wide range of scientific and engineering applications beyond the current domain.

## Supporting information

S1 Appendix Appendices (A, B, C & D). (PDF)

S1 Data Data. (ZIP)
