# Supplementary material for: Phase II monitoring of process variability in multichannel profiles
Source: PLoS One. 2025 Dec 12;20(12):e0337707. doi: 10.1371/journal.pone.0337707 (PMC12700403; doi:10.1371/journal.pone.0337707)
Supplement: S1 Appendix — (PDF) [file pone.0337707.s001.pdf]

Appendix A: Pseudocode for Data Generation

The Pseudocode based on the provided MATLAB code for data generation is given as follows:

```
Initialize l,k, p, m, n, mu, corr, sigma0, shift, and basis

For l = 1 to 6 do:
    For kk = 1 to k do:
        For i = 1 to p do:
            For j = 1 to p do:
%Calculate the covariance matrix using a correlation structure
                sigma (i, j, kk) = kk* corr^ (abs (i - j));
            End For
        End For
%Generate multivariate normal random samples for eta (for each channel)
        eta (: kk, :) = mvnrnd (mu, sigma(:,:,kk), m)';
     End For
% Apply shift to the covariance matrix
    sigma1 = (shift(l)*sigma0) + sigma;
% Define u as a range from 0 to 1 with n grid points
    u = linspace (0, 1, n);
    Y = zeros (p, numel(u), m);
    For i = 1 to m do:
       For j = 1 to numel(u) do:
           A = zeros(p, 1);
               For kk = 1 to k do:
                   A = A + (basis(j, kk)*eta(:, kk, i));
               End For
           Y(:, j, i) = A;
       End For
    End For
    For i = 1 to m do:
         x(:, :, i) = repmat(mu, 1, n) + Y(:, :, i);
    End For
End For
```

Appendix B: Pseudocode for MFPCA

The Pseudocode for MFPCA is as below:

```
Initialize meanX, covX, temp:
% Compute the mean
For i = 1 to m do:
    For j = 1 to p do:
      meanX(j, h) = mean(x(j, h, :));
    End For
End For
%Compute the covariance matrix
For h = 1 to n do:
    For l = 1 to n do:
        For i = 1 to m do:
            For j = 1 to p do:
                temp = temp +(1/m)*(x(j,h,i)- meanX(j,h))*(x(j,l,i)-meanX(j,l));
            End For
        End For
        covX(h, l) = temp;
```

```
    End For
End For

%Perform Eigenvalue Decomposition on the covariance matrix
 Compute [V, D] = eig(covX);
%V contains the eigenvectors, D contains the eigenvalues
 D = diag(D);
% Ensure the eigenvalues are real and avoid small negative values
 D = max(1e-5, real(D));
% Ensure eigenvectors are real
 V = real(V);
% Sort eigenvalues in descending order and sort corresponding eigenvectors
 [~,SO] = sort(D,'descend');
 D = D(SO);
 V = V(:, SO);
%Compute the cumulative sum of the sorted eigenvalues
Cumsum = cumsum(D);
percent = Cumsum /sum(D);
%Determine the number of principal components that explain at least 90% of the
variance
d = find(percent > 0.9, 1, 'first');
% Select the eigenvectors corresponding to the top 'd' principal components
final_v = V(:, 1:d);
```

Appendix C: Pseudocode for Computation of the Chart Statistic

The Pseudocode for computation of the chart statistic is provided as

```
Initialize m,d,eps,s,F,T,lambda,sig_k,isig_k,temp 1,eta0,im:

For im = 1 to m do:
%Project the data onto the selected principal components (final_v)
  temp1(:,:,im) = temp1(:, :,im)+(x(:,:,im)-meanX)*final_v;
End For
For k = 1 to d do:
%Compute the covariance for the k-th principal component

    For im = 1 to m do:
        sig_k(:,:,k)= sig_k(:,:,k)+(temp1(:,k,im))*(temp1(:,k,im))';
    End For
    sig_k(:,:,k)= sig_k(:,:,k)/m;
%Compute the inverse of the covariance matrix
    isig_k(:, :, k) = inv(sig_k(:, :, k));
End For

While im < m do:
    im = im + 1;

%Compute eta0 for the current sample by projecting onto the final_v PCs
    eta0(:,:,im)= x(:,:,im)*final_v;
End While

For im = 2 to m do:
   For k = 1 to d do:
     s{im, k} = (1-lambda)*s{im -1,k} + lambda*(eta0(:,k,im)*eta0(:,k,im)');
```

```
     F(im, k)=trace(s{im, k})-log(det(s{im, k})+eps)-p;
   End For
  T(im, 1)=sum(F(im,:));
End For
```

Appendix D: Pseudocode for ARL and SDRL computation of the proposed control chart

The Pseudocode for computing ARL and ADRL of the proposed control chart based on simulation is as follows:

```
Initialize UCL,RL,L:
For l = 1 to L do:
    Set RL(i,1)= 0;

    For run = 1 to 50 do:
        Set Loop = 0;
        Set jj = 0;
        While Loop == 0 do:
            Increment jj by 1;
            Generate the chart statistic
            If statistic(jj) > UCL then:
                Set Loop = 1;      %Exit loop
                Set RL(run, 1) = jj;  %Record the run length when UCL is
exceeded
            End If
        End While
    End For
    Set ARL(l,:)=mean(RL);
    Set SDRL(l,:)=std(RL) ;
End For
```
