# Supplementary material for: Phase II monitoring of process variability in multichannel profiles
Source: PLoS One. 2025 Dec 12;20(12):e0337707. doi: 10.1371/journal.pone.0337707 (PMC12700403; doi:10.1371/journal.pone.0337707)
Supplement: S1 Data — (ZIP) [file pone.0337707.s002.zip › Data/readme.docx]

**Simulation Study Data**

The numerical results are obtained with 10000 simulation runs in this paper. The number of channels is equal to. According to the generic IC model that, the in-control mean functions are zero. The processes are sampled on a grid of equidistant points in Figure 3 represents the process of computing ARL in the proposed control chart.

We consider where are the first four non-constant and Fourier basis functions with a base period of 0.5, and follows a *p*-dimensional multivariate normal distribution with **mean vector 0** and **covariance matrix.** The correlation is considered as =0.8. The dimension of the covariance matrix in the multichannel profile relates to the interrelationship among the channels.

Mean=[0;0;0;0]

=[1 0.8 0.64 0.512;0.8 1 0.8 0.64;0.64 0.8 1 0.8; 0.512 0.64 0.8 1]

=[2 1.6 1.28 1.024;1.6 2 1.6 1.28;1.28 1.6 2 1.6; 1.024 1.28 1.6 2]

=[3 2.4 1.92 1.536; 2.4 3 2.4 1.92; 1.92 2.4 3 2.4;1.536 1.92 2.4 3]

=[4 3.2 2.56 2.048;3.2 4 3.2 2.56;2.56 3.2 4 3.2;2.048 2.56 3.2 4]

Fourier basis=[4.973798e-01 1.9371663 9.635073e-01 1.7526134; 9.635073e-01 1.7526134 1.688656e+00 1.0716536; 1.369094e+00 1.4579373 1.996053e+00 0.1255810;1.688656e+00 1.0716536 1.809654e+00 -0.8515586; 1.902113e+00 0.6180340 1.175571e+00 -1.6180340;1.996053e+00 0.1255810 2.506665e-01 -1.9842294; 1.964575e+00 -0.3747626 -7.362491e-01 -1.8595530; 1.809654e+00 -0.8515586 -1.541026e+00 -1.2748480; 1.541026e+00 -1.2748480 -1.964575e+00 -0.3747626; 1.175571e+00 -1.6180340 -1.902113e+00 0.6180340;

7.362491e-01 -1.8595530 -1.369094e+00 1.4579373; 2.506665e-01 -1.9842294 -4.973798e-01 1.9371663; -2.506665e-01 -1.9842294 4.973798e-01 1.9371663; -7.362491e-01 -1.8595530 1.369094e+00 1.4579373; -1.175571e+00 -1.6180340 1.902113e+00 0.6180340; -1.541026e+00 -1.2748480 1.964575e+00 -0.3747626; -1.809654e+00 -0.8515586 1.541026e+00 -1.2748480; -1.964575e+00 -0.3747626 7.362491e-01 -1.8595530; -1.996053e+00 0.1255810 -2.506665e-01 -1.9842294; -1.902113e+00 0.6180340 -1.175571e+00 -1.6180340; -1.688656e+00 1.0716536 -1.809654e+00 -0.8515586; -1.369094e+00 1.4579373 -1.996053e+00 0.1255810;

-9.635073e-01 1.7526134 -1.688656e+00 1.0716536; -4.973798e-01 1.9371663 -9.635073e-01 1.7526134; -4.898425e-16 2.0000000 -9.796851e-16 2.0000000; 4.973798e-01 1.9371663 9.635073e-01 1.7526134; 9.635073e-01 1.7526134 1.688656e+00 1.0716536; 1.369094e+00 1.4579373 1.996053e+00 0.1255810; 1.688656e+00 1.0716536 1.809654e+00 -0.8515586; 1.902113e+00 0.6180340 1.175571e+00 -1.6180340; 1.996053e+00 0.1255810 2.506665e-01 -1.9842294; 1.964575e+00 -0.3747626 -7.362491e-01 -1.8595530; 1.809654e+00 -0.8515586 -1.541026e+00 -1.2748480; 1.541026e+00 -1.2748480 -1.964575e+00 -0.3747626; 1.175571e+00 -1.6180340 -1.902113e+00 0.6180340; 7.362491e-01 -1.8595530 -1.369094e+00 1.4579373; 2.506665e-01 -1.9842294 -4.973798e-01 1.9371663; -2.506665e-01 -1.9842294 4.973798e-01 1.9371663; -7.362491e-01 -1.8595530 1.369094e+00 1.4579373; -1.175571e+00 -1.6180340 1.902113e+00 0.6180340; -1.541026e+00 -1.2748480 1.964575e+00 -0.3747626; -1.809654e+00 -0.8515586 1.541026e+00 -1.2748480; -1.964575e+00 -0.3747626 7.362491e-01 -1.8595530; -1.996053e+00 0.1255810 -2.506665e-01 -1.9842294; -1.902113e+00 0.6180340 -1.175571e+00 -1.6180340; -1.688656e+00 1.0716536 -1.809654e+00 -0.8515586; -1.369094e+00 1.4579373 -1.996053e+00 0.1255810; -9.635073e-01 1.7526134 -1.688656e+00 1.0716536; -4.973798e-01 1.9371663 -9.635073e-01 1.7526134; -9.796851e-16 2.0000000 -1.959370e-15 2.0000000];

**Illustrative Example**

Out-of-control multi-channel profile data under a shift (scenario 1) induced in covariance matrix is generated till the proposed statistic exceeds the *UCL*.

| Observation (*i*) | 1 | 2 | 3 | 4 | 5 | 6 | 7 | 8 | 9 | 10 |
| --- | --- | --- | --- | --- | --- | --- | --- | --- | --- | --- |
| statistic | 43.26 | 49.78 | 53.61 | 54.02 | 55 | 59.35 | 61.11 | 62.5 | 63.8 | 68 |
| Observation (*i*) | 11 | 12 | 13 | 14 | 15 | 16 | 17 | 18 |  |  |
| statistic | 53.61 | 58.87 | 64.28 | 70.15 | 72.47 | 75.69 | 79.08 | 81.24 |  |  |

**Case Study: An Application** **in Multi-Operation Forging Process**

We demonstrate the practical implementation of the proposed method through a multi-operation forging process, using tonnage.RDATA which is attached in supplementary data.

The length of each profile channel is 200. A sample of 220 multichannel profiles was collected under different experimental settings. The sample includes 151 in-control profiles collected under the in-control production condition (C1Nr-C4Nr CSV files) and a group of 69 OC profiles (C1F4r-C4F4r CSV files). These OC profiles are measured when one part is missing in the piercing station.

**CSV Files**

** **C1Nr, C2Nr, C3Nr, C4Nr:** Tonnage profiles for **in-control condition** from four sensors (one per column of the forging machine).

**** C1F4r, C2F4r, C3F4r, C4F4r:** Tonnage profiles for **OC conditions (piercing fault)** from those same four sensors.

| Observation (*i*) | 1 | 2 | 3 | 4 | 5 | 6 | 7 | 8 | 9 |
| --- | --- | --- | --- | --- | --- | --- | --- | --- | --- |
| statistic | 60.12 | 53.61 | 58.87 | 70.15 | 64.28 | 82.68 | 75.25 | 71.74 | 87.09 |
